# Trimethylamine-N-oxide promotes osteoclast differentiation and oxidative stress by activating NF-κB pathway

**DOI:** 10.18632/aging.205869

**Published:** 2024-05-27

**Authors:** Yangyang Zhao, Chizhen Wang, Fei Qiu, Jing Liu, Yujuan Xie, Zhengkun Lin, Jianquan He, Jian Chen

**Affiliations:** 1 Department of Rehabilitation, Zhongshan Hospital of Xiamen University, School of Medicine, Xiamen University, Xiamen, China; 2 School of Medicine, Xiamen University, Xiamen, China; 3 Xiamen Humanity Rehabilitation Hospital, Xiamen, China

**Keywords:** trimethylamine-N-oxide, osteoclast, NF-κB, inflammation, reactive oxygen species

## Abstract

Background: Senile osteoporosis may be caused by an imbalance in intestinal flora and oxidative stress. Trimethylamine-N-oxide (TMAO), a metabolite of dietary choline dependent on gut microbes, has been found to be significantly increased in osteoporosis. However, the role of TMAO in bone loss during osteoporosis remains poorly understood. In this study, we examined the impact of TMAO on osteoclast differentiation and bone resorption in an *in vitro* setting.

Methods: Osteoclast differentiation was induced by incubating RAW 264.7 cells in the presence of Receptor Activator for Nuclear Factor-κB Ligand (RANKL) and macrophage-stimulating factor (M-CSF). Flow cytometry, TRAP staining assay, CCK-8, and ELISA were employed to investigate the impact of TMAO on osteoclast differentiation and bone resorption activity *in vitro*. For mechanistic exploration, RT-PCR and Western blotting were utilized to assess the activation of the NF-κB pathway. Additionally, protein levels of secreted cytokines and growth factors were determined using suspension array technology.

Results: Our findings demonstrate that TMAO enhances RANKL and M-CSF-induced osteoclast formation and bone resorption in a dose-dependent manner. Mechanistically, TMAO triggers the upregulation of the NF-κB pathway and osteoclast-related genes (NFATc1, c-Fos, NF-κB p65, Traf6, and Cathepsin K). Furthermore, TMAO markedly elevated the levels of oxidative stress and inflammatory factors.

Conclusions: In conclusion, TMAO enhances RANKL and M-CSF-induced osteoclast differentiation and inflammation in RAW 264.7 cells by activating the NF-κB signaling pathway. These findings offer a new rationale for further academic and clinical research on osteoporosis treatment.

## INTRODUCTION

Osteoporosis (OP) is a common metabolic skeletal disorder defined by the deterioration of bone matrix and weakened bone strength, leading to heightened bone fragility and vulnerability to fractures [1]. Osteoporosis can be caused by anything that decreases bone formation or increases bone resorption. Osteoclasts, which can be differentiated from monocyte/macrophage lineages, mainly control bone resorption and play a crucial role in maintaining bone balance [2]. The Macrophage Colony-Stimulating Factor (M-CSF) is the main cytokine that promotes the formation of osteoclasts by interacting with its receptor, c-fms, leading to the maturation of osteoclast precursors into fully functional osteoclasts [3]. The secretion of the Receptor Activator of Nuclear Factor-κB Ligand (RANKL) by osteoblasts, bone marrow stromal cells [4], and lymphocytes [5], members of the tumor necrosis factor superfamily, plays a crucial role in bone metabolism. Once RANKL binds to the nuclear factor (NF)-κB receptor activator (RANK), it triggers the recruitment of tumor necrosis factor receptor-associated factor 6 (TRAF6), leading to the activation of mitogen-activated protein kinase (MAPK) and transcription factors [6, 7]. Subsequently, the downstream signals c-Fos and NFATc1, crucial for osteoclast differentiation, are activated to promote osteoclastogenesis [8]. Recent studies suggest that the gut microbiota may influence bone metabolism regulation, playing a significant role in the development and progression of osteoporosis. Trimethylamine-N-oxide (TMAO), a metabolite of dietary choline that relies on gut microbes, has been strongly linked to various diseases. Choline, a compound that includes trimethylamine and belongs to the phosphatidylcholine group, undergoes metabolism by gut microbes to generate an intermediate compound known as TMA. Subsequently, TMA is efficiently oxidized by hepatic monooxygenases (FMO1 and FMO3) to yield TMAO [9]. The primary sources of this nutrient are dairy products, fish, shrimp, red meat, wheat, and beans. In 2011, Wang et al. first proposed the potential harm of TMAO to human health [10]. Nowadays there is convincing evidence suggesting an association between TMAO and inflammation [11, 12]. Osteoporosis is currently acknowledged as an inflammatory condition. The findings of Li et al. indicate that decreased levels of TMAO could potentially decelerate bone loss by mitigating the inflammatory response in older mice with low bone mass [13]. Furthermore, the results of a case-control study revealed a substantial elevation in serum TMAO levels among postmenopausal women who experienced hip fractures compared to the control group [14]. Wang and colleagues found that TMAO enhances the differentiation of osteoclasts and leads to bone loss through the NF-κB signaling pathway [15]. However, this correlation needs to be further verified, and the correlation between TMAO and the level of inflammation in osteoclast differentiation is still unknown.

As a result, the objective of this study was to investigate how TMAO might potentially enhance the differentiation and bone resorption functions of osteoclasts, while also examining the molecular mechanisms that drive its effects on oxidative and inflammatory responses. Our analysis focused on reactive oxygen metabolites and the levels of inflammatory cytokine expression in osteoclasts exposed to TMAO-stimulated RAW 264.7 macrophages.

## MATERIALS AND METHODS

### Materials

**Figure 1:**
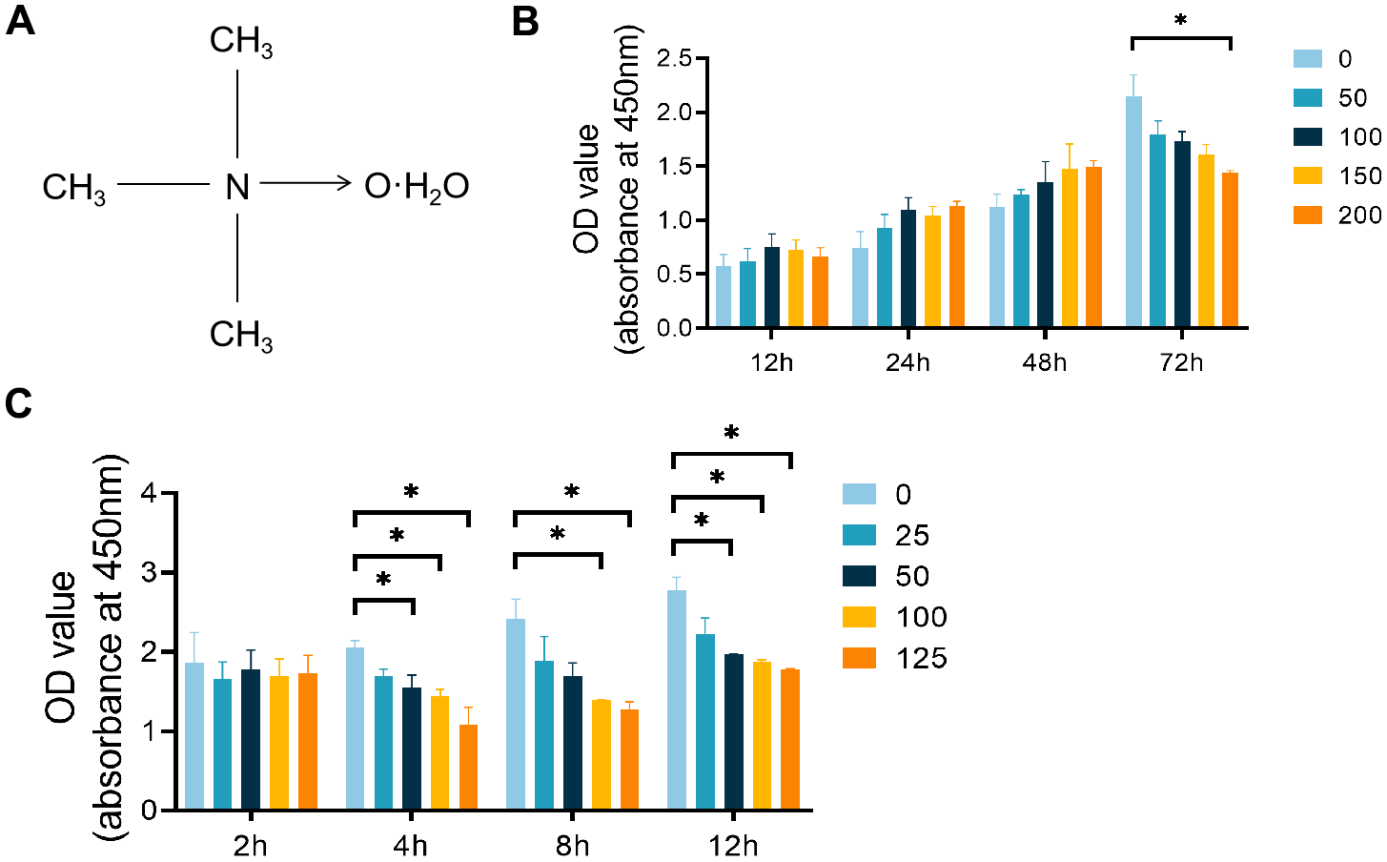
**Effects of TMAO and PDTC on the viability of RAW 264.7 cells.** (**A**) Molecular structure of TMAO. (**B**) The cell viability of RAW 264.7 cells after treatment with different concentrations of TMAO for 12, 24, 48, and 72 h was evaluated by CCK-8. The cell viability of RAW 264.7 cells decreased significantly at 72 h with 200 μM TMAO treatment. (**C**) The cell viability of RAW 264.7 cells after treatment with different concentrations of PDTC for 2, 4, 8, and 12 h was evaluated by CCK-8. The cell viability of RAW 264.7 cells decreased significantly at 4 h with 50 μM PDTC treatment. * *p* < 0.05, ** *p* < 0.01.

TMAO (Figure 1A) and pyrrolidine dithiocarbamate (PDTC, an NF-κB inhibitor) were purchased from Topscience (Shanghai, China). Fetal bovine serum (FBS) was purchased from ExCell Bio (Shanghai, China). Dulbecco’s modified Eagle’s medium (DMEM) and streptomycin were obtained from VivaCell (Israel). The Cell Counting Kit-8 (CCK-8) was acquired from Dojindo Molecular Technologies (Kumamoto, Japan). Recombinant M-CSF and sRANK Ligand were sourced from Peprotech (Cranbury, NJ, USA). The Tartrate-Resistant Acid Phosphatase (TRAP) Stain Kit was purchased from Solarbio (Beijing, China). The Beta Crosslaps (β-CTx) enzyme-linked immunosorbent assay (ELISA) Kit was obtained from FineTest (Wuhan, China).

### Cell culture

In this study, the murine leukemia macrophage cell RAW 264.7 was used to stimulate the differentiation of osteoclasts. The RAW 264.7 cell line was provided by iCell Bioscience Inc. (Shanghai, China) and cultured in high glucose DMEM supplemented with 10% FBS. The cells were harvested and utilized for experimentation. Cell cultures were maintained in a humidified incubator with 5% CO_2_. To investigate the involvement of the NF-κB pathway in TMAO-induced osteoclast formation, RAW 264.7 cells were pretreated with PDTC before exposure to 100 μmol/L TMAO.

### Cytotoxicity assays

The Cell Counting Kit-8 was used to evaluate the viability of RAW 264.7 cells when exposed to TMAO and PDTC, following the instructions provided by the manufacturer. Cells were plated in 96-well plates at a concentration of 4×10^3^ cells/well with varying concentrations of TMAO (0-200 μmol/L) for 12 hours, 1 day, 2 days, or 3 days. Additionally, cells were plated at a density of 1×10^5^ cells/well with different concentrations of PDTC (0-125 μmol/L) for 2 hours, 4 hours, 8 hours, or 12 hours. Following incubation with the CCK-8 solution for 1 hour, the optical density was then assessed at 450 nm using a Multiskan SkyHigh full-wavelength microplate reader by Thermo Fisher Scientific, located in St. Louis, MO, USA.

### Osteoclastogenesis assays carried out *in vitro*

RAW 264.7 cells were cultured with 10 ng/mL of M-CSF and 50 ng/mL of RANKL to induce the generation of osteoclasts. The control group consisted of untreated RAW 264.7 cells. Other groups were treated with TMAO and PDTC, individually. Four days later, the cells were stained for TRAP activity according to the manufacturer’s guidelines. Osteoclasts were identified as TRAP-positive polynuclear cells with more than 3 nuclei (TRAP^+^ MNCs) using a Fluorescent Inverted microscope (Olympus, Tokyo, Japan).

### Bone resorption activity analysis

The amount of β-CTx serves as an indicator of the bone resorption activity of osteoclasts, making it a crucial biochemical marker for bone resorption [16]. The production of β-CTx was measured by suspending RAW 264.7 cells in DMEM with 10% fetal bovine serum and adding culture supernatants after 4 days of culturing with 50 ng/mL RANKL, 10 ng/mL M-CSF, and TMAO/PDTC. The β-CTx concentration in culture supernatants was quantified using an immunocapture enzyme assay kit in an ELISA format. The optical density was determined at 450 nm with a Multiskan SkyHigh full-wavelength enzyme label (Thermo Fisher Scientific, St. Louis, MO, USA).

### Real-time PCR analysis

4×10^5^ RAW 264.7 cells were plated in each well of a 6-well plate. Following a 3-day treatment with RANKL (50 ng/mL) and M-CSF (10 ng/mL), the four experimental groups received TMAO (100 μM) and PDTC (25 μM) for 24 hours. Total RNA extraction was carried out using the RNApure Tissue and Cell Kit (CWBIO, Beijing, China). 1 μg of single-stranded cDNA was isolated with reverse transcriptase from Bio-Rad (Hercules, CA, USA). Real-time PCR was conducted with iTaq Universal SYBR Green Supermix (Bio-Rad, Hercules, CA, USA), and the results were analyzed with the LightCycler 96 system (Basel, Switzerland). Primers were designed targeting specific mouse sequences for the experiments: NFATc1 (forward: TCCAAAGTCATTTTCGTGGA, reverse: CTTTGCTTCCATCTCCCAGA), c-Fos (forward: CGGGTTTCAACGCCGACTA, reverse: TTGGCACTAGAGACGGACAGA), TRAF6 (forward: AGGAATCACTTGGCACGACACTTG, reverse: TCGCACGGACGC AAAGCAAG), Cathepsin K (forward: CGAAAAGA GCCTAGCGAACA, reverse: TGGGTAGCAGCAGAAACTTG), NF-κB p65 (forward: CGGATTGAAGAAAAACG, reverse: TTGAAAAGGCATAGGGC), GAPDH (forward: AACTTTGGCATTGTGGAAGG, reverse: ACACATTGGGGGTAGGAACA). The data were analyzed using the 2-ΔΔCT method, and all results were adjusted to the GAPDH level.

### Protein preparation and Western blot analysis

After treatment with either TMAO or PDTC in a 6-well plate according to the instructions, RAW 264.7 cells were lysed using RIPA buffer (NCM Biotech, Suzhou, China) for extracting total protein on ice. The overall protein concentration was assessed using a BCA protein detection kit from NCM Biotech in Suzhou, China. The proteins in the samples were then isolated using an 8% SDS-PAGE electrophoresis system. The isolated proteins were transferred to a PVDF membrane (0.22 μM, PALL, New York, NY, USA) and subsequently incubated at room temperature in 5% skim milk for 60 minutes for sealing. After gentle shaking overnight, membranes were incubated with primary antibodies, washed thrice with TBST (Meilunbio, Dalian, China), and then exposed to corresponding secondary antibodies (CWBIO, Beijing, China) for 1 hour. The detection of antibody-antigen complexes was performed using ECL reagent (NCM Biotech, Suzhou, China). Primary antibodies used in the study included TRAF6 (ab33915), NF-κB p65 (ab32536), c-Fos (ab222699), and GAPDH (ab181602) purchased from Abcam (San Diego, CA, USA). Cathepsin K (Ctsk, sc-48353) and NFATc1 (sc-7294) were provided by Senta (OR, USA).

### Intracellular ROS assay

ROS detection was performed on RAW 264.7 cells by stimulating them with M-CSF and RANKL for 3 days. Subsequently, the cells were exposed with TMAO or PDTC and incubated in serum-free medium at 37° C with 10 μM DCFH-DA (Yeasen, Shanghai, China) for 30 minutes. The fluorescence was measured using the Spark Multifunctional label Instrument (Tecan, Männedorf, Switzerland) at an excitation wavelength of 488 nm and an emission wavelength of 525 nm. Subsequently, flow cytometry (FCM) analysis was conducted using a Beckman Coulter instrument from Bria, CA, USA to assess the relative ROS levels in the cells, with data being analyzed using Kaluza software.

### Inflammatory factor assay

RAW 264.7 cells were suspended in DMEM containing 10% fetal bovine serum and received cell supernatant after 4 days of culture in the presence of 50 ng/mL RANKL, 10 μg/μL M-CSF and TMAO or PDTC. The levels of cytokines in the culture supernatants were examined using the Bio-Plex murine 23-Plex Panel Kit from Bio-Rad Laboratories, Inc., following the provided instructions. This system enables the simultaneous detection of cytokines in a 96-well filter plate, as detailed in the study by Niu et al. [17].

### Statistical analysis

Data analysis was performed using GraphPad Prism 8.0 software. The results represent three independent experiments and are presented as mean ± standard deviation (SD). The significance of differences was determined using one-way ANOVA followed by Dunnett’s multiple comparison test. A difference at *p < 0.05 was considered significant and that at **p < 0.01 was considered highly significant.

## RESULTS

### Effects of TMAO and PDTC on the viability of RAW 264.7 cells

To avoid any harmful effects of TMAO, we employed a CCK-8 kit to assess its cytotoxicity and impact on RAW 264.7 cell proliferation. The cell count notably declined after 72 hours of exposure to 150 μM TMAO (Figure 1B). Subsequently, we limited the TMAO concentration to a maximum of 100 μM for the following experiments. To establish the most effective pretreatment time and PDTC dose, a CCK-8 assay was conducted. A 4-hour exposure to 50 μM PDTC resulted in a significant decrease in RAW 264.7 cell numbers (Figure 1C). As a result, we proceeded with a maximum PDTC concentration of 25 μM and a 2-hour pretreatment time for further analyses.

### TMAO promoted osteoclast differentiation

**Figure 2:**
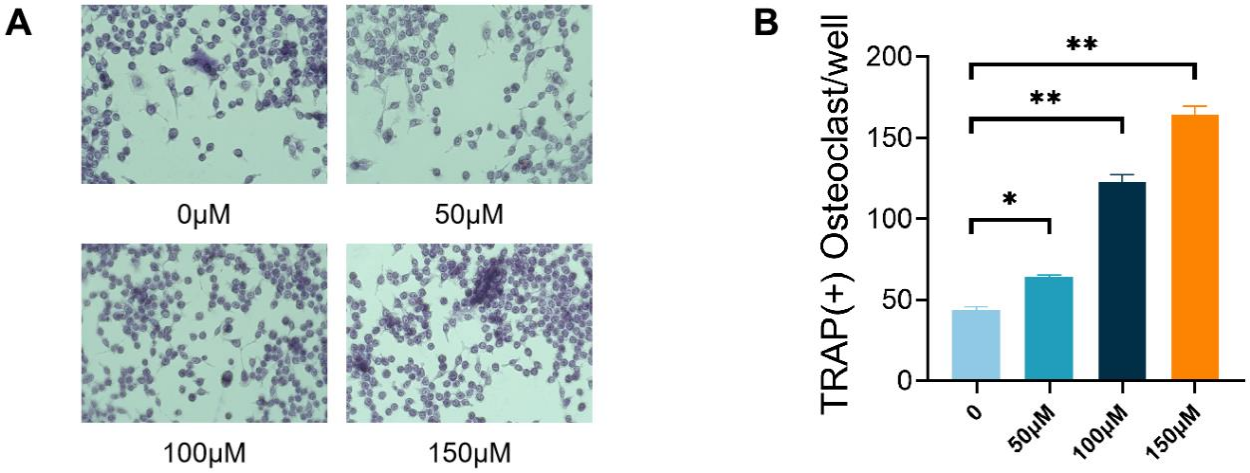
**TMAO promoted RANKL-induced osteoclast differentiation.** (**A**) RAW 264.7 cells were cultured for 4 d with RANKL (50 ng/mL) and M-CSF (10 ng/mL) in the presence of varying concentrations of TMAO and then stained for TRAP activity. Representative photomicrographs were taken under a light microscope (magnification ×40). (**B**) TRAP-positive cells containing more than three nuclei were counted as osteoclasts; ** *p* < 0.01.

To study how TMAO affects RANKL-induced osteoclast formation, RAW 264.7 cells treated with RANKL were exposed to varying TMAO concentrations and analyzed for osteoclast development. The RANKL-treated RAW 264.7 cells showed the presence of TRAP^+^ MNCs. Upon exposure to TMAO, there was a progressive increase in the quantity of osteoclasts induced by RANKL, with the effect being dependent on the concentration of TMAO (Figure 2A, 2B).

### TMAO enhanced RANKL-induced osteoclast-associated gene expression

**Figure 3:**
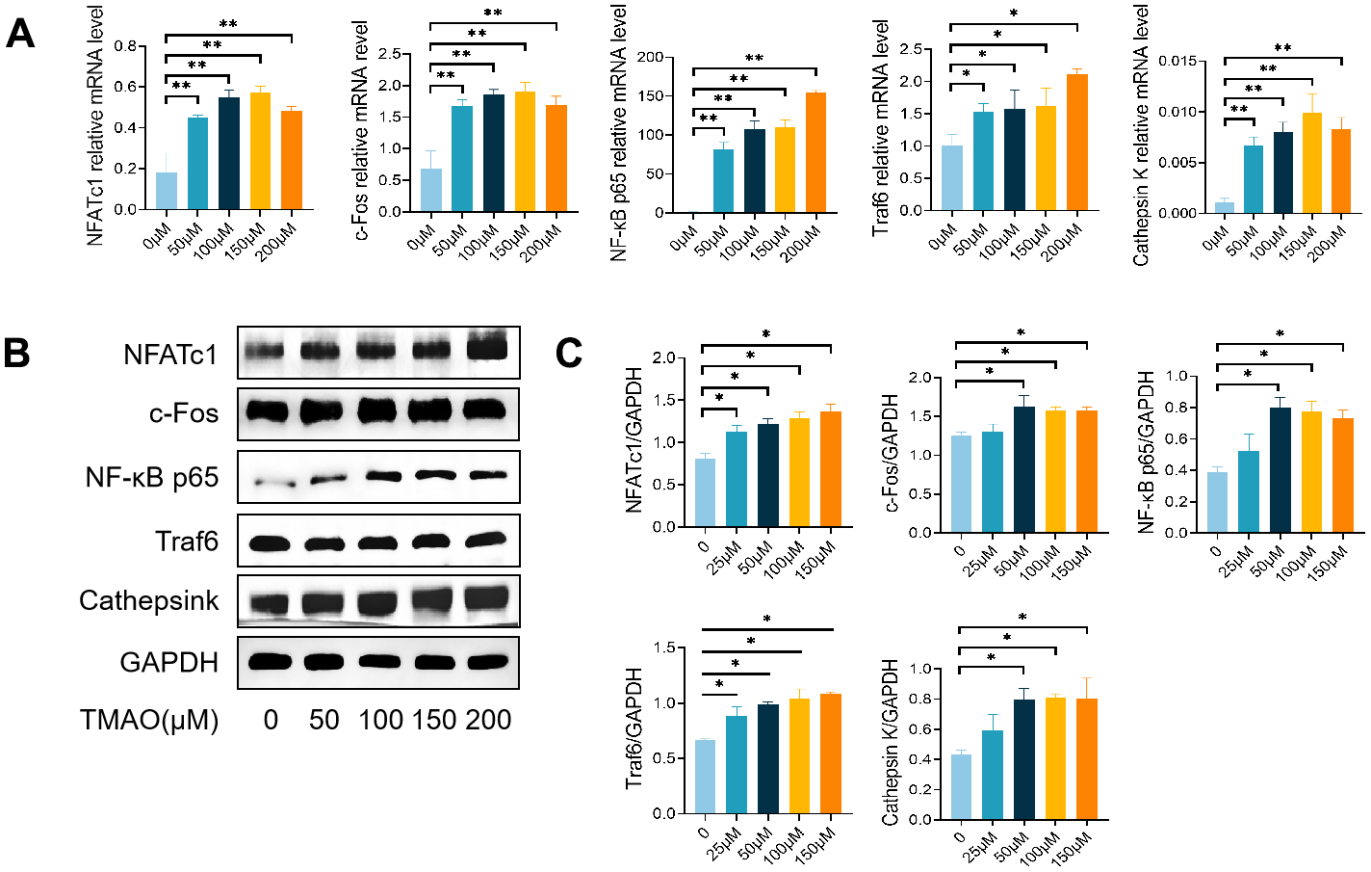
**TMAO enhanced RANKL-induced osteoclast-associated gene expression.** (**A**) TMAO enhanced osteoclast gene expression (n = 4 per group) as examined by real-time PCR. (**B**, **C**) TMAO enhanced NFATc1, c-Fos, Cathepsin K, NF-κB p65, and Traf6 protein expression as evaluated by Western blot (n = 3 per group). * *p* < 0.05, ** *p* < 0.01.

In order to study how TMAO affects osteoclast differentiation induced by RANKL, we examined the levels of mRNA and proteins of genes related to osteoclasts in the presence and absence of TMAO. The levels of NFATc1, c-Fos, NF-κB p65, Traf6, and Ctsk were observed to rise in a dose-dependent fashion when exposed to TMAO in the context of osteoclast formation (Figure 3). These findings suggest that TMAO promotes osteoclastogenesis and bone resorption.

### TMAO enhanced RANKL-induced activation of NF-κB

**Figure 4:**
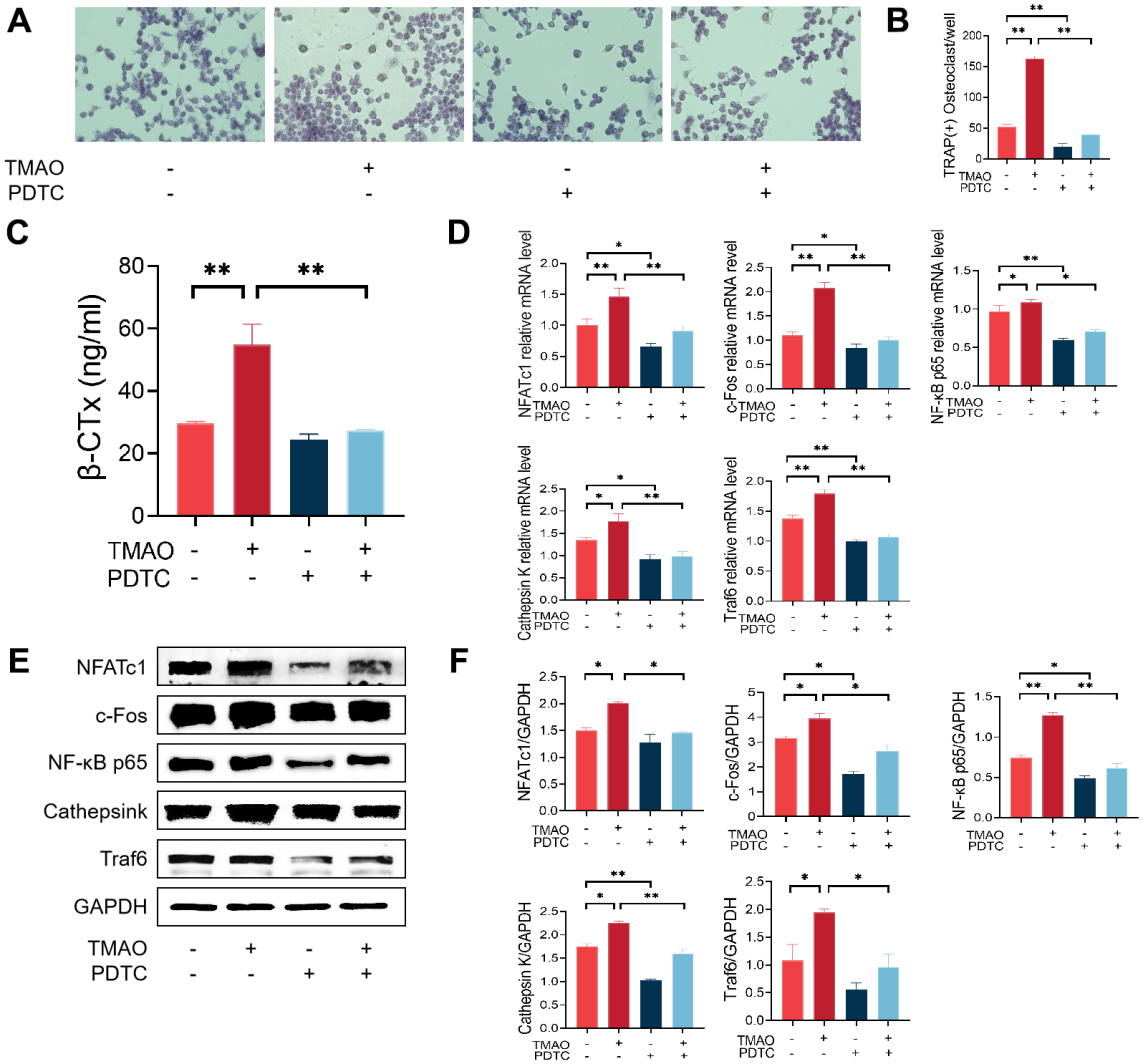
**Inhibition of the NF-κB signaling pathway with PDTC reversed the effect of TMAO on osteoclast differentiation.** (**A**, **B**) RAW 264.7 cells were pretreated with 25 μM PDTC for 2 h before being cultured with or without 100 μM TMAO. The number of TRAP-positive osteoclasts increased under 100 μM TMAO and decreased with PDTC pretreatment with or without TMAO. (magnification ×40). (**C**) The expression of β-CTx was up-regulated under 100 μM TMAO and suppressed by PDTC with or without TMAO treatment evaluated by ELISA (n = 3 per group). (**D**) The expression of osteoclast-specific genes was up-regulated under 100 μM TMAO and suppressed by PDTC with or without TMAO treatment evaluated by real-time PCR (n = 4 per group). (**E**, **F**) The protein expression levels of NFATc1, c-Fos, Cathepsin K, NF-κB p65, and Traf6 were promoted under 100 μM TMAO and suppressed by PDTC with or without TMAO treatment as examined by Western blot (n = 3 per group). * *p* < 0.05, ** *p* < 0.01.

Pyrrolidine dithiocarbamate (PDTC) is a potent inhibitor of nuclear factor-κB (NF-κB). We proved that TMAO could induce osteoclast differentiation. PDTC was then employed to evaluate the inhibitory effect of TMAO on osteoclast differentiation, thereby demonstrating the crucial involvement of NF-κB signaling in TMAO-induced osteoclast differentiation. Initially, the number of osteoclasts increased after TRAP staining in the presence of 100 μM TMAO, and PDTC pretreatment reversed this increase irrespective of the presence or absence of TMAO (Figure 4A, 4B). Subsequently, the average β-CTx levels significantly increased following treatment with 100 μM TMAO (Figure 4A, 4B). Subsequently, the average β-CTx level significantly increased after treatment with 100 μM TMAO (Figure 4C). In addition, the levels of β-CTx increased significantly when 100 μM TMAO treatment was added after 2 h of PDTC pretreatment compared with PDTC treatment group. Third, the higher levels of Traf6, NF-κB p65, Ctsk, c-Fos, and NFATc1 were all up-regulated in the presence of 100 μM TMAO and were subsequently down-regulated with PDTC pretreatment (Figure 4D). The protein expression of Traf6, NFATc1, Ctsk, c-Fos, and NF-κB p65 showed an increase when treated with 100 μM TMAO, as anticipated, and decreased with PDTC treatment (Figure 4E, 4F).

### TMAO increased ROS levels during osteoclast differentiation

**Figure 5:**
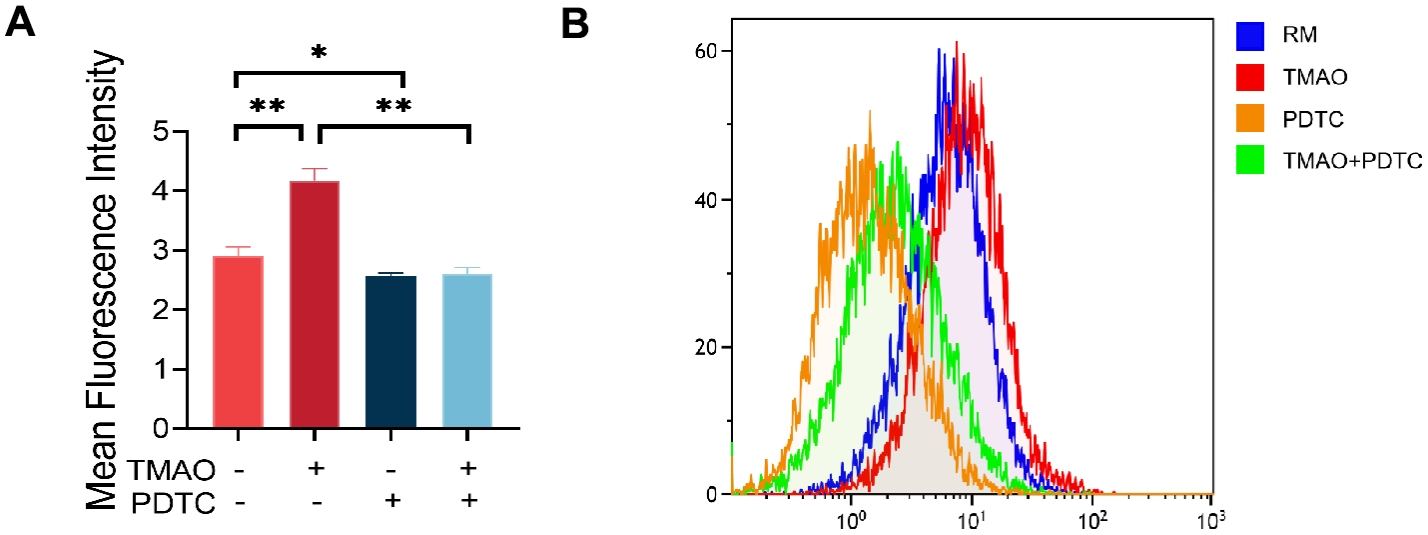
**TMAO increased ROS levels in RAW 264.7 cells during osteoclast differentiation.** (**A**) RAW 264.7 cells were stimulated with RANKL and M-CSF for 4 days and then probed with 10 μM DCFH-DA for 30 min after being treated with TMAO or/and PDTC. TMAO treatment led to increased fluorescence intensity of ROS in RAW 264.7 cells, and PDTC treatment decreased TMAO-induced ROS generation (n = 5 per group). * *p* < 0.05, ** *p* < 0.01. (**B**) The ROS production in RAW 264.7 cells after being treated with TMAO or/and PDTC was detected by FCM. RM: RANKL and M-CSF.

Research has indicated that TMAO is linked to oxidative stress and the excessive production of ROS in an inflammatory condition. To investigate the effect of ROS on TMAO-mediated osteoclast differentiation, we assessed the levels of ROS in RAW 264.7 cells exposed to TMAO. The findings revealed that TMAO treatment led to a rise in the fluorescence intensity of ROS in RAW 264.7 cells (Figure 5A, 5B).

### TMAO enhanced inflammation level during osteoclast differentiation

**Figure 6:**
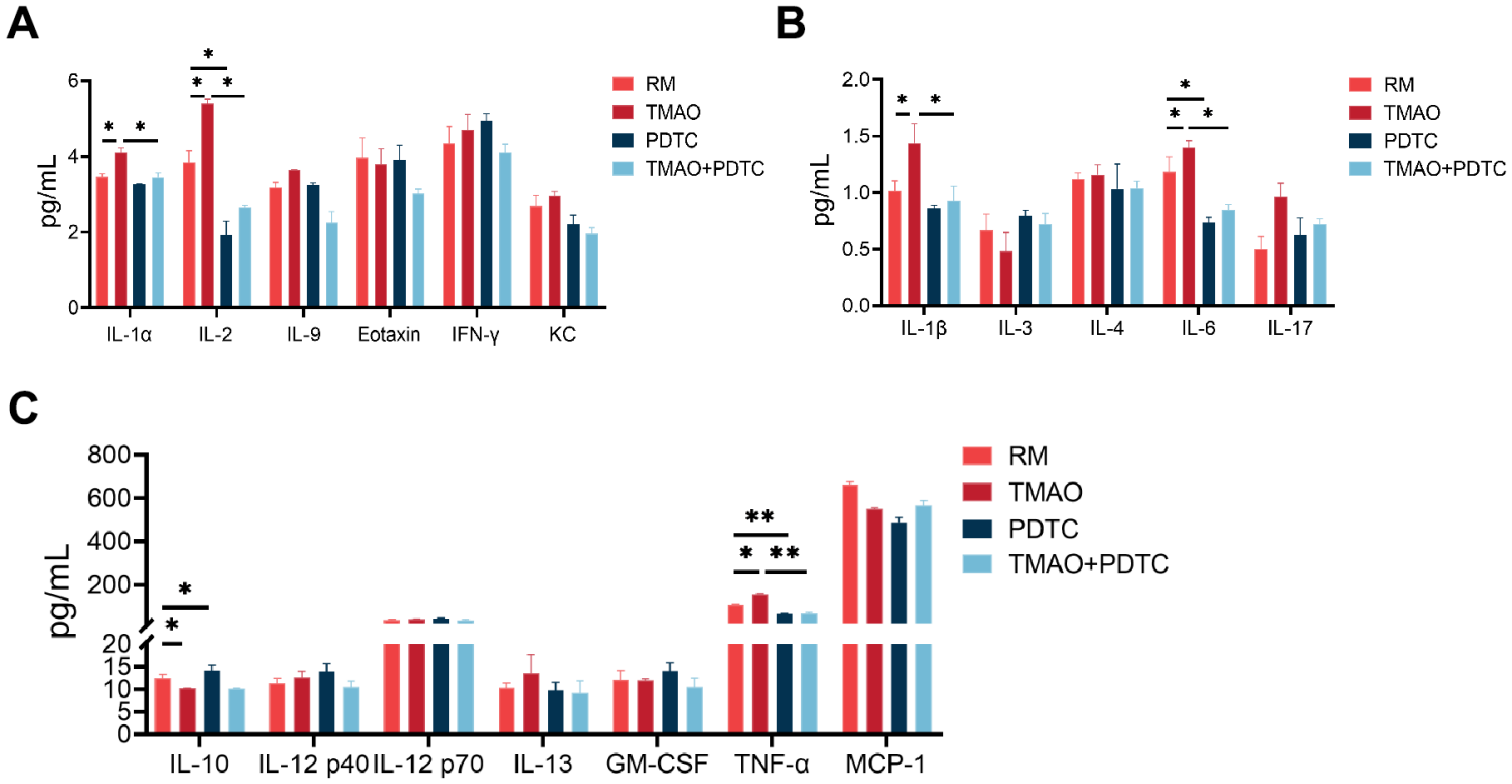
**TMAO enhanced inflammation level during osteoclast differentiation, analyzed by the Bio-Plex murine 23-Plex Panel Kit (Bio-Rad Laboratories).** (**A**) IL-1α and IL-2 were elevated in the culture in the existence of TMAO stimulation. (**B**, **C**) IL-1β, IL-6, and TNF-α were also elevated, whereas IL-10 were significantly reduced in the TMAO cultures. RM: RANKL and M-CSF. * *p* < 0.05, ** *p* < 0.01.

Suspension array techniques were utilized to analyze the secretion levels of various cytokines and growth factors in culture supernatants treated with TMAO and/or PDTC. The findings indicated that IL-1α and IL-2 levels were increased in the culture following exposure to TMAO stimulation (Figure 6A). Additionally, levels of TNF-α, IL-1β, and IL-6 were found to be higher, while IL-10 was significantly lower in the TMAO cultures (Figure 6B, 6C). However, the expression of these cytokines was significantly reduced in the cocultures by PDTC and TMAO. Furthermore, IL-5, MIP-1α, MIP-1β, IFN-c, and RANTES concentration cannot be detectable in the culture (data not shown).

## DISCUSSION

Our current research showed that *in vitro*, TMAO has the ability to stimulate the differentiation of osteoclasts by activating the NF-κB signaling pathway. Furthermore, we observed that TMAO can increase inflammation levels during osteoclast differentiation. These results are vital for deciphering the ways in which TMAO plays a role in bone loss associated with osteoporosis.

With aging, excessive activation of osteoclasts leads to accelerated bone loss and markedly enhances the vulnerability to various bone diseases including osteoporosis and osteoarthritis [18]. Osteoporosis is linked to a higher risk of falls, with fractures being the most severe complication that significantly impacts the quality of life of patients. Research has indicated that higher levels of TMAO, a metabolite of dietary choline produced by gut microbes, are linked to an increased likelihood of hip fractures in elderly individuals. This suggests that increased levels of TMAO could be a contributing factor to bone loss and the risk of fractures among older individuals [19]. Nevertheless, there is currently scarce knowledge regarding its impact on bone loss in osteoporosis.

A recent study demonstrated that a concentration of 400 μM TMAO notably reduced the viability of BMMs [15]. However, treating bovine aortic endothelial cells (BAE-1) with 10 mM TMAO did not result in any detrimental impact on cell viability [20]. The concentration threshold of TMAO was identified as 150 μM in this study, according to the findings of the *in vitro* cell viability test. Consequently, various TMAO concentrations were chosen for the subsequent experiments. The utilization of divergent dosages implied that TMAO can exert varying levels of toxicity on distinct cell lines. Alternatively, in the experiment involving the concentration gradient of TMAO, 100 μM TMAO promoted osteoclast differentiation and related protein expression better. By collecting serum samples of osteoporosis patients and healthy subjects, Lin et al. found that the serum TMAO level of osteoporosis patients increased significantly. They also looked into how TMAO affects cell activity in a controlled environment and found that TMAO concentrations of 50 and 100 μM did not have a notable impact on cell viability. However, concentrations of TMAO ranging from 200 to 600 μM significantly hindered cell growth in a manner that depended on both time and dosage [21]. Therefore, we set the concentration of 100 μM in the current study and this concentration is appropriate.

RAW 264.7 cells, frequently employed as osteoclast precursors, exhibit the ability to differentiate into osteoclasts upon stimulation by RANKL and M-CSF. The results of our first investigation showed that RANKL and M-CSF induced the formation of TRAP-positive osteoclasts and bone resorption in pre-osteoclastic RAW 264.7 cells, a process that was further stimulated by TMAO at a concentration of 100 μM (Figures 2, 3). After that, we explored the mechanisms by which TMAO affects the differentiation and functionality of osteoclasts.

The importance of NF-κB signaling in osteoclast formation has been shown to be essential [22]. Thus, the inhibition of NF-κB activation could potentially have a significant impact on the process of osteoclast formation. We used PDTC, an inhibitor of NF-κB, to block the effect of TMAO on osteoclast differentiation. The findings indicated that TMAO can promote osteoclast formation, which is significantly inhibited by PDTC. Subsequently, we investigated the mechanisms underlying the promotion of osteoclast formation by TMAO. We identified NFATc1 and c-Fos as crucial regulators in the process of osteoclast formation [23]. Furthermore, NFATc1 can upregulate the expression of other transcription factors linked to osteoclastogenesis, such as c-fos, NF-κB, and NFATc2, thereby increasing its efficacy [24]. Subsequently, we examined the impact of TMAO on NFATc1 and c-Fos, revealing that TMAO upregulated the mRNA and protein expression of NFATc1 and c-Fos in RANKL-stimulated RAW 264.7 cells. Moreover, prior research has demonstrated a close association between NFATc1 activation, Traf6 recruitment, and NF-κB activation. It has also been shown that NFATc1 can induce osteoclasts to produce specific proteins, such as Ctsk and TRAP, which are crucial for bone matrix absorption [25]. Therefore, we investigated the mRNA and protein levels of Traf6, NF-κB p65, and Ctsk. The findings revealed that TMAO treatment significantly increased the expression of these genes during RANKL-induced osteoclast formation. The results suggest that TMAO plays a role in the creation of osteoclasts by boosting the expression of NFATc1 and other related transcription factors.

**Figure 7:**
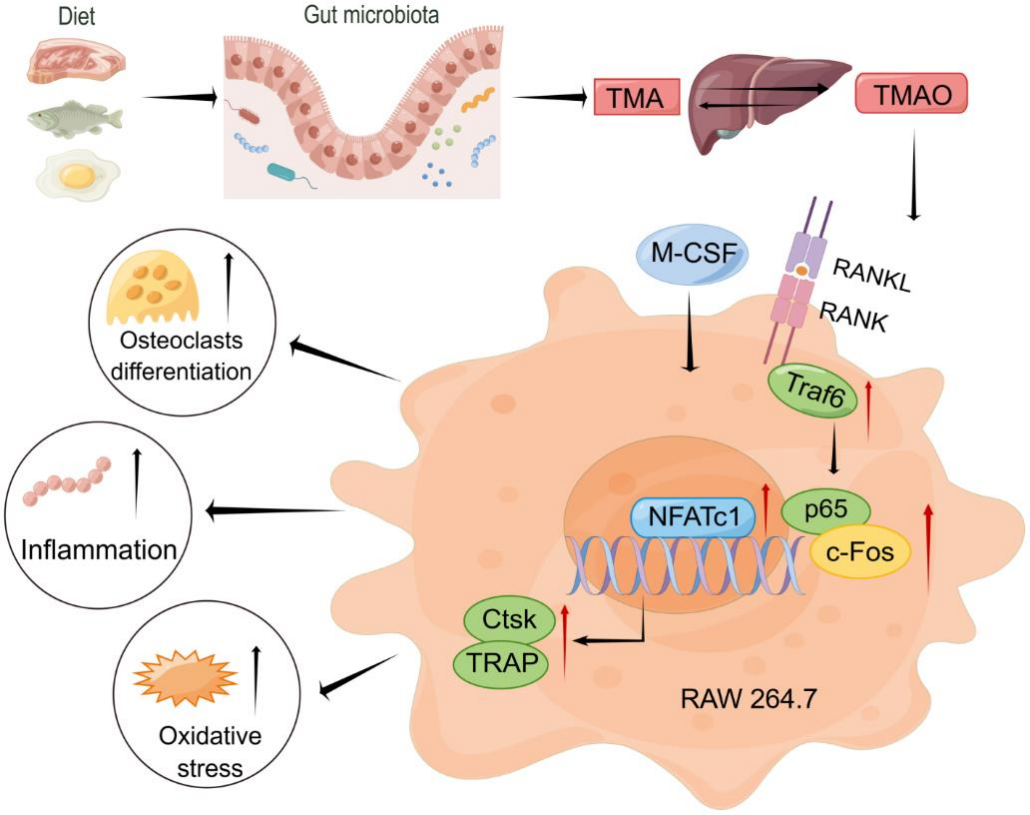
Working model drawn by Figdraw illustrating the promotion mechanism of TMAO on RANKL-induced osteoclast differentiation.

Inflammation and oxidative stress are deemed significant factors in the development of chronic diseases, with macrophages serving as key immune cells that modulate inflammation through the generation of inflammatory proteins as well as reactive oxygen species (ROS), cytokines, and proinflammatory chemokines [26]. Udagawa and his colleagues verified the origin of osteoclasts as monocytes/macrophages for the first time in 1990 [27]. The fusion and multinucleation of macrophages are crucially confirmed to be necessary for the development of osteoclasts in regulating bone mass [28]. The current research demonstrated that TMAO enhanced the differentiation of RAW 264.7 cells into osteoclasts *in vitro* by activating NF-κB, a finding that was corroborated by PDTC treatment. Furthermore, TMAO elevated inflammatory markers during the process. This aligns with the notion that TMAO has been observed to be a risk factor for a range of chronic inflammatory conditions [10, 29]. Senescence is characterized by elevated levels of oxidative stress and increased production of ROS. Recent studies have indicated that oxidative stress is involved in the regulation of osteoclast formation [30, 31]. As people get older, levels of ROS tend to rise, which then stimulates osteoclast activity and causes bone degradation [32, 33]. The latest findings suggest that TMAO, which is triggered by RANKL and M-CSF, may help reduce oxidative stress by activating the NF-κB signaling pathway. This, in turn, results in enhanced bone loss and increased formation of osteoclasts in macrophage RAW 264.7 cells (Figure 7).

TMAO, a byproduct produced by gut bacteria, has been found to be closely linked to cardiovascular disease and other health conditions. Recently, gut microbiota (GM) has received increasing attention in the pathways regulating bone metabolism. Qiao et al. [34] evaluated the characteristics of GM and fecal metabolomics in osteoporosis rat models. There was a notable difference in the abundance of GM between the osteoporosis group and the control group. Additionally, osteoporosis caused significant changes in fecal metabolites. The results suggested a correlation between changes in GM abundance, fecal metabolites, and bone loss. Furthermore, Lin et al. [21] discovered that OP patients had significantly higher TMAO levels in their serum compared to the healthy group. Conversely, findings from a prospective study suggest that TMAO could potentially help prevent bone mineral density (BMD) loss during weight loss. This indicates that the impact of changes in plasma TMAO levels on BMD changes may be influenced by dietary fat [35].

Aging in humans is associated with chronic inflammation, and the excessive accumulation of senescent cells in tissues can have a detrimental effect on regenerative capacity and contribute to the development of a pro-inflammatory environment [36, 37]. Moreover, chronic inflammation has been established as a risk factor for morbidity and mortality in the elderly population [38]. Recent evidence suggests that age-related alterations in human gut microbiota can trigger immune aging and inflammation, potentially worsening neurodegenerative diseases, metabolic syndrome, susceptibility to infection, and other conditions. Ultimately, this impacts the overall quality of life for older individuals [39]. TMAO functions as a derivative of the gut microbiome. Brunt et al. confirmed that circulating TMAO levels are elevated in older adults compared to younger adults [40]. According to Chen and colleagues, elevated TMAO levels in mice led to higher levels of pro-inflammatory cytokines and decreased levels of anti-inflammatory cytokines [41]. Numerous hypotheses exist regarding the mechanism by which TMAO promotes aging, but consensus is currently lacking. One hypothesis suggests that TMAO inhibits SIRT1 expression, leading to increased oxidative stress and activation of the p53/p21/Rb pathway. Consequently, this leads to heightened acetylation of P53 and P21, as well as reduced phosphorylation of Rb, CDK2, and cyclinE1, thereby accelerating endothelial cell apoptosis and vascular aging [42]. These findings align with our research indicating an association between high TMAO levels with elevated oxidative stress and inflammation. However, further validation is required using human subjects. On another note, age-related decline in physical function often leads to reduced physical activity among older individuals who play an important role in maintaining good health through exercise. Argyridou et al.’s study demonstrated that engaging in chronic moderate physical activity is linked with lower TMAO levels [43]. In addition, TMAO is a risk factor for age-related cognitive decline that impairs learning and memory function, and exercise can reverse this process [44]. Although the aging process is universal, progressive, gradual and irresistible, aging management targeting human gut microbiota is a new way to promote health and anti-aging. Regulating the human gut microbiota effectively has emerged as a novel approach to maintaining the health of the elderly.

This study explores the impact of TMAO on osteoporosis development and progression, offering novel avenues for investigating potential treatment targets, particularly in individuals with chronic inflammation. Our results show that TMAO has the ability to stimulate the formation of osteoclasts by triggering the NF-κB signaling pathway in a laboratory setting. Oxidative stress and inflammation levels are upregulated in this process. This is consistent with the results of *in vivo* experiments by Zhang et al. [17]. The main reason for osteoporosis is the increased activity of osteoclasts compared to osteoblasts. While we have demonstrated the ability of TMAO to induce bone loss through promoting osteoclast differentiation and activating NF-κB *in vitro*, additional research is essential to elucidate the impact of TMAO in the human *in vivo* setting. It is important to measure TMAO levels in humans to reflect real-life physiological conditions accurately. It is essential to carry out rigorous clinical trials to confirm the relationship between TMAO and BMD or bone turnover markers in individuals with osteoporosis. Even with these constraints, our research demonstrated that TMAO promotes the differentiation of osteoclasts and increases levels of inflammation, both of which are associated with the NF-κB signaling pathway.

## CONCLUSIONS

In summary, our laboratory experiments showed that TMAO influenced the differentiation of macrophages into osteoclasts and heightened the inflammatory response. Furthermore, the increase in inflammatory markers helped move NF-κB to the nucleus, resulting in higher levels of osteoclast-specific protein expression. It is plausible that targeting TMAO could be a promising approach for treating osteoporosis, particularly in individuals with chronic inflammatory conditions.
